# Intramedullary Nailing Versus Modular Megaprosthesis in Pathological Humeral Fractures: A 10-Year Retrospective Comparative Study

**DOI:** 10.3390/diseases14060218

**Published:** 2026-06-18

**Authors:** Giuseppe Rovere, Federica Messina, Cesare Meschini, Maria Serena Oliva, Matteo Caredda, Fernando De Maio, Pasquale Farsetti, Giulio Maccauro, Antonio Ziranu

**Affiliations:** 1 Orthopaedic Unit, Department of Surgical Sciences, University Hospital of Tor Vergata, 00133 Rome, Italy; 2 Department of Clinical Sciences and Translational Medicine, Tor Vergata University, 00133 Rome, Italy; demaio@med.uniroma2.it (F.D.M.); farsetti@uniroma2.it (P.F.); 3 Orthopaedic and Traumatology Department, S. Spirito Hospital, Lungotevere in Sassia 1, 00193 Rome, Italy; federicamessina695@gmail.com; 4 Orthopaedics and Traumatology Department, Università Cattolica del Sacro Cuore, 00168 Rome, Italy; cesare.meschini@gmail.com (C.M.); giulio.maccauro@policlinicogemelli.it (G.M.); antonio.ziranu@unicatt.it (A.Z.); 5 Orthopaedic and Traumatology Department, Casa di Cura Villa Bianca, 73100 Lecce, Italy; 6 Department of Orthopedics, Ospedale Isola Tiberina-Gemelli Isola, 00186 Rome, Italy; mariaserena.oliva@gmail.com (M.S.O.); matteocaredda92@gmail.com (M.C.); 7 Orthopaedics and Trauma Surgery Unit, Department of Ageing, Neurosciences, Head-Neck and Orthopaedics Sciences, Fondazione Policlinico Universitario Agostino Gemelli IRCCS, 00168 Rome, Italy

**Keywords:** humeral metastases, pathological fractures, intramedullary nailing, megaprosthesis, orthopedic oncology, functional outcomes, MSTS, QuickDASH, WOSI, survival analysis

## Abstract

Background: Pathological fractures of the humerus secondary to metastatic disease represent a significant cause of pain, disability, and reduced quality of life in oncologic patients. Surgical management aims to restore stability, reduce pain, and allow early mobilization. However, the optimal strategy between intramedullary nailing and modular megaprosthesis remains debated, particularly in relation to functional outcomes and long-term results. Methods: A retrospective observational study was conducted on 48 patients treated for pathological or impending humeral fractures between January 2015 and January 2025. Twenty-six patients underwent intramedullary nailing (IMN group), while twenty-two were treated with tumor resection and modular megaprosthesis reconstruction (MP group). Functional outcomes were assessed using the Musculoskeletal Tumor Society (MSTS) score, Quick Disabilities of the Arm, Shoulder and Hand (QuickDASH), and Western Ontario Shoulder Instability Index (WOSI) at 1, 12, 24, 36, and 60 months, and at 10 years. Complications and overall survival were also analyzed. Results: Intramedullary nailing demonstrated significantly superior early functional outcomes, with higher MSTS scores at 1 month (78% vs. 63%, *p* < 0.001) and lower QuickDASH scores in the first 24 months (*p* = 0.002). WOSI scores also favored IMN in the early postoperative period (*p* = 0.004). Megaprosthesis showed a slower initial recovery but a progressive improvement over time, reaching comparable functional outcomes at long-term follow-up (*p* > 0.05). The overall complication rate was similar between groups (*p* = 0.28), although periprosthetic infections occurred only in the MP group. Survival analysis did not show significant differences between groups (*p* = 0.74). Conclusions: Both intramedullary nailing and modular megaprosthesis represent effective surgical options for pathological and impending humeral fractures. Intramedullary nailing provides faster early functional recovery, whereas megaprosthetic reconstruction offers a durable reconstructive solution for extensive proximal lesions. Functional outcomes progressively converged between the two techniques approximately 2–3 years after surgery. Mid-term outcomes up to five years appeared comparable, suggesting that surgical decision-making should be individualized according to lesion characteristics, tumor biology, expected survival, and functional demands.

## 1. Introduction

Bone metastases represent a common and clinically relevant complication in oncologic patients, often leading to severe pain, functional impairment, and reduced quality of life. Among long bones, the humerus is the second most frequently affected site after the femur [1,2,3].

Pathological fractures and impending fractures, as defined by Mirels criteria, are associated with significant disability of the upper limb and a marked loss of autonomy [4]. Conservative treatment has shown limited efficacy in this setting, particularly in terms of pain control and functional recovery, making surgical management the preferred option in most cases [5].

The primary goals of surgery are to provide immediate mechanical stability, reduce pain, and enable early mobilization, thereby improving overall quality of life. Two main surgical strategies are commonly employed: intramedullary nailing, typically indicated for diaphyseal lesions, and resection with reconstruction using modular megaprosthesis, generally reserved for proximal lesions with joint involvement [6].

Intramedullary nailing is less invasive and allows rapid functional recovery, making it particularly suitable for patients with limited life expectancy. Conversely, megaprosthetic reconstruction enables complete tumor resection and improved local disease control, especially in patients with longer survival expectancy [7,8].

Recent studies have emphasized the importance of individualized surgical planning based on patient prognosis, tumor characteristics, and anatomical considerations. Rovere et al. demonstrated the relevance of tailored strategies in proximal humeral metastases [9], while El Motassime et al. highlighted the progressive functional improvement observed in patients treated with megaprosthetic reconstruction [10].

Advancements in implant technology and oncologic reconstruction techniques have improved outcomes and implant survival, with megaprostheses showing reliable long-term results in metastatic bone disease [7,8]. However, complications such as infection remain a concern, particularly in complex reconstructions [8].

Despite these developments, there is still a lack of studies directly comparing intramedullary nailing and modular megaprosthesis with long-term follow-up. This represents a relevant gap in the literature, as the choice of surgical strategy significantly influences both early functional recovery and long-term outcomes.

The aim of this study is to compare intramedullary nailing and modular megaprosthesis in the treatment of pathological and impending humeral fractures, focusing on functional outcomes, complications, and long-term results over a 10-year follow-up period.

## 2. Materials and Methods

This retrospective observational study was conducted at a single tertiary referral center between January 2015 and January 2025. The study was performed in accordance with the principles of the Declaration of Helsinki and reported following the PROCESS guidelines for observational studies.

A total of 48 consecutive patients treated for pathological or impending fractures of the humerus were included in the study. The cohort consisted of 19 males (39.6%) and 29 females (60.4%), with a mean age of 66 years (range 41–87 years).

At presentation, 45 patients (93.7%) had a complete pathological fracture, while 3 patients (6.3%) presented with an impending fracture, defined according to Mirels criteria (score ≥ 9) [4]. The Mirels scoring system evaluates the risk of pathological fracture based on four variables (anatomical site, pain, lesion type, and lesion size), generating a score ranging from 4 to 12 points. Lesions with a score ≥ 9 are considered at high risk of fracture and are generally regarded as candidates for prophylactic surgical stabilization.

Patients with multiple myeloma and multiple lesions were included if at least one humeral lesion required surgical stabilization.

### 2.1. Inclusion Criteria

- Age ≥ 18 years.
- Pathological fracture or impending fracture of the humerus.
- Mirels score ≥ 9 for impending fractures.
- Minimum follow-up of 6 months.

### 2.2. Exclusion Criteria

- Primary bone tumors.
- Traumatic fractures.
- Revision surgery.
- Incomplete clinical or follow-up data.

Patients were divided into two groups based on the surgical treatment performed:

- Intramedullary nailing group (IMN group, *n* = 26; 54.2%)
  Patients underwent fixation with locked intramedullary nails. This approach was preferentially adopted for diaphyseal or metadiaphyseal lesions without extensive joint involvement.
- Megaprosthesis group (MP group, *n* = 22; 45.8%)
  Patients underwent en bloc tumor resection followed by reconstruction with a modular megaprosthesis. This technique was mainly reserved for proximal humeral lesions with articular compromise or extensive bone destruction.

The choice of surgical technique was based on a multidisciplinary evaluation, considering lesion location, extent of bone involvement, patient prognosis, and functional demands.

#### Surgical Technique and Rehabilitation Protocol

Megaprosthetic reconstruction was performed using modular proximal humeral tumor prostheses. Stem fixation was achieved using cemented or uncemented techniques according to bone quality, residual bone stock, and surgeon preference. Particular attention was paid to soft-tissue balancing and reattachment of the remaining rotator cuff and deltoid structures whenever feasible.

All patients followed a standardized postoperative rehabilitation protocol. Passive shoulder mobilization was initiated during the early postoperative period, followed by progressive active-assisted and active range-of-motion exercises after wound healing. Strengthening exercises were gradually introduced according to pain tolerance, functional recovery, and oncological status. Patients undergoing megaprosthetic reconstruction generally required a longer rehabilitation period due to the extent of bone resection and soft-tissue reconstruction.

### 2.3. Primary Outcomes

Functional outcomes and quality of life were assessed using validated scoring systems:

- Musculoskeletal Tumor Society (MSTS) score
  Evaluates pain, function, emotional acceptance, and independence. Scores range from 0 to 30 and are expressed as a percentage (0–100%).
- Quick Disabilities of the Arm, Shoulder and Hand (QuickDASH) score
  Patient-reported outcome measure assessing upper limb disability (0 = no disability; 100 = maximum disability).
- Western Ontario Shoulder Instability Index (WOSI)
  Assesses shoulder-related quality of life across four domains, with scores ranging from 0 (best) to 2100 (worst).

### 2.4. Secondary Outcomes

- Postoperative complications included infection, mechanical failure, nerve injury. Infections were classified as superficial, deep, or periprosthetic according to clinical findings and microbiological evidence. For the purposes of this study, only deep and periprosthetic infections requiring prolonged antibiotic therapy and/or surgical treatment were included in the analysis.
- Local disease control (recurrence in megaprosthesis group).
- Overall survival.

Patients were evaluated at standardized time points:

- 1 month (T0);
- 12 months;
- 24 months;
- 36 months;
- 60 months;
- 10 years.

Clinical and functional assessments were performed at each follow-up visit.

Continuous variables were expressed as mean ± standard deviation (SD), while categorical variables were reported as frequencies and percentages.

The normality of data distribution was assessed using the Shapiro–Wilk test. Comparisons between groups were performed using Student’s *t*-test for continuous variables and the chi-square test or Fisher’s exact test for categorical variables.

Longitudinal functional outcomes (MSTS, QuickDASH, and WOSI) were analyzed using repeated measures analysis of variance (ANOVA) to evaluate changes over time and interaction effects between treatment groups.

Survival analysis was performed using the Kaplan–Meier method, and differences between groups were assessed using the log-rank test.

Statistical analysis was performed using IBM SPSS Statistics for Windows, Version 28.0 (IBM Corp., Armonk, NY, USA).

## 3. Results

A total of 48 patients were included in the study, with no significant differences between the intramedullary nailing (IMN) and megaprosthesis (MP) groups in terms of baseline characteristics. Baseline demographic and clinical characteristics of the study population are summarized in Table 1. Treatment allocation reflected the anatomical characteristics of the lesions. Intramedullary nailing was preferentially used for diaphyseal and metadiaphyseal lesions without major articular involvement, whereas megaprosthetic reconstruction was generally selected for proximal lesions associated with extensive bone destruction and joint compromise.

The mean age was 66 years (range 41–87), with no statistically significant difference between groups (*p* = 0.62). Sex distribution was comparable (*p* = 0.71).

At presentation, 45 patients (93.7%) had a complete pathological fracture, while 3 patients (6.3%) presented with an impending fracture (Mirels ≥ 9).

Two patients affected by multiple myeloma presented with multiple skeletal lesions requiring surgical stabilization.

**Figure 1:**
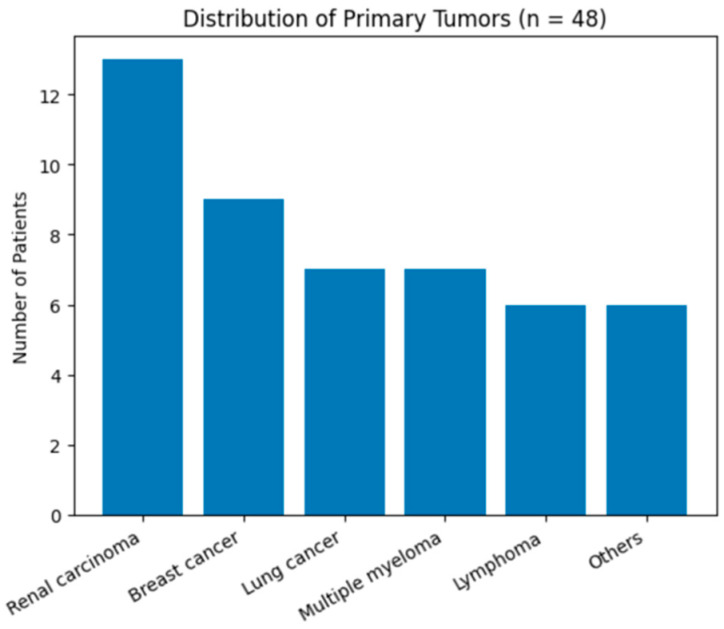
Distribution of primary tumors in the study population (*n* = 48). Bar chart illustrating the frequency of primary tumor types leading to humeral metastases.

The distribution of primary tumors did not significantly differ between the two groups (*p* = 0.68) (Figure 1).

**Figure 2:**
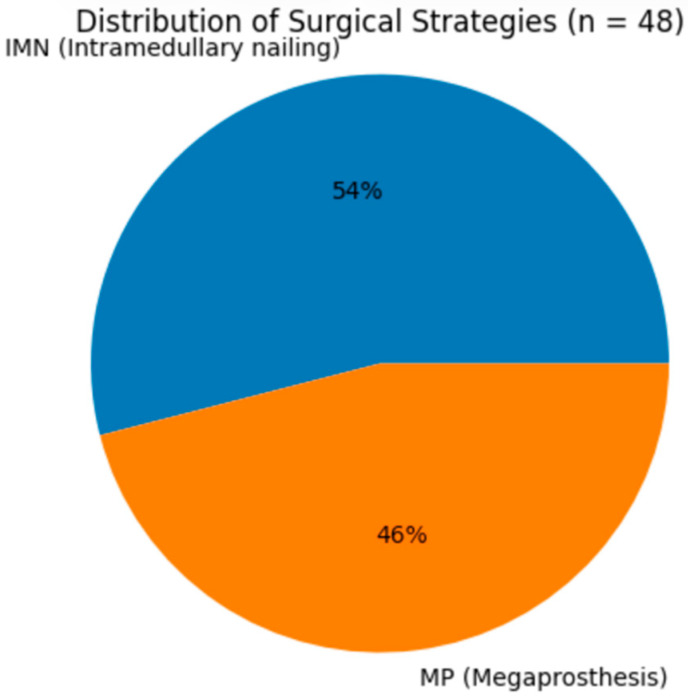
Distribution of surgical strategies in the study cohort (*n* = 48). Pie chart showing the proportion of patients treated with intramedullary nailing (IMN) and modular megaprosthesis (MP).

Twenty-six patients (54.2%) underwent intramedullary nailing, while 22 patients (45.8%) were treated with tumor resection and modular megaprosthesis reconstruction (Figure 2).

No intraoperative complications were reported in either group.

Postoperative complications are summarized in Table 2.

The overall complication rate did not significantly differ between groups (*p* = 0.28). Although infections were observed exclusively in the MP group, this difference did not reach statistical significance (*p* = 0.18).

No cases of mechanical failure requiring revision surgery were observed during the follow-up.

During the follow-up period:

- Twenty-eight patients (58.3%) died;
- Twenty patients (41.7%) were alive at 5 years;
- Four patients (8.3%) were alive at 10 years.
  Accordingly, functional outcome analyses at 5 and 10 years were based on the surviving patients available for clinical assessment at those time points.

**Figure 3:**
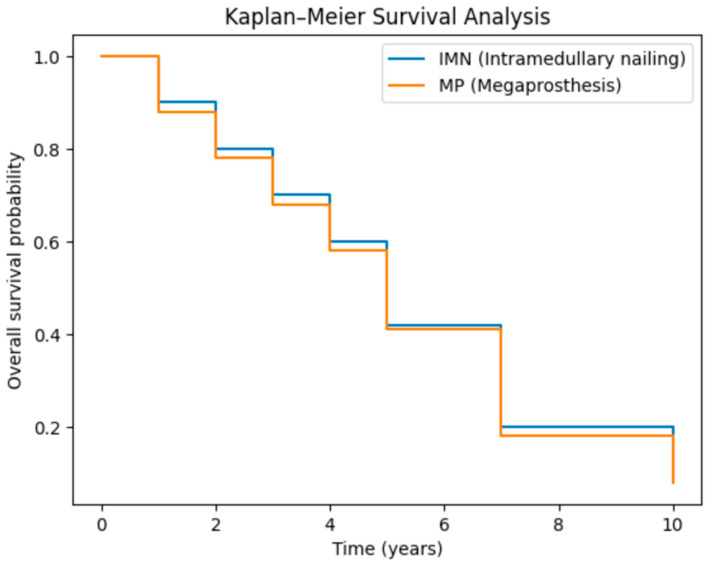
Kaplan–Meier survival curves for the intramedullary nailing and megaprosthesis groups. Kaplan–Meier survival analysis showing comparable overall survival between the two treatment groups, with no statistically significant differences observed over time (log-rank test, *p* = 0.74).

Kaplan–Meier survival analysis showed no statistically significant differences between the IMN and MP groups (log-rank test, *p* = 0.74), (Figure 3), indicating that survival was primarily related to the underlying oncological disease rather than the surgical technique.

At 1 month postoperatively, the IMN group demonstrated significantly higher MSTS scores compared to the MP group (78% vs. 63%, *p* < 0.001).

At 12 and 24 months, the IMN group maintained superior functional outcomes (*p* = 0.01). However, this difference progressively decreased over time.

**Figure 4:**
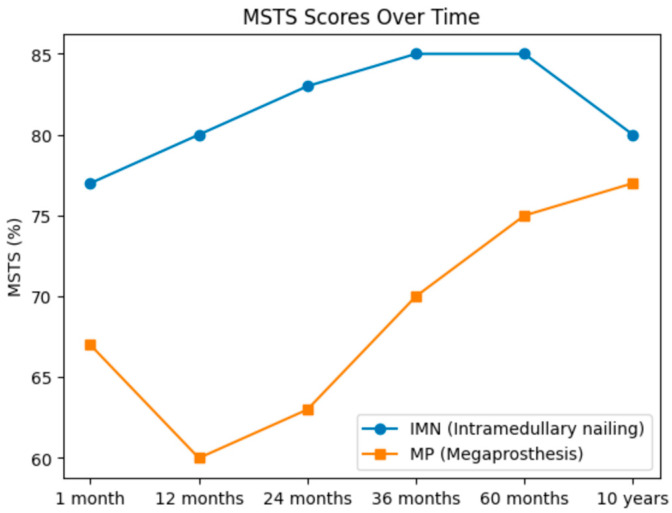
Longitudinal MSTS scores over time in the intramedullary nailing and megaprosthesis groups. Line graph demonstrating superior early functional performance in the intramedullary nailing group, followed by a gradual convergence of outcomes between groups at long-term follow-up.

At long-term follow-up (10 years), no statistically significant difference was observed between the two groups (*p* = 0.67), with both treatments achieving comparable functional outcomes over time (Figure 4).

Patients treated with intramedullary nailing showed significantly lower disability scores during early follow-up.

At 1 month, QuickDASH scores were significantly better in the IMN group (28 vs. 45, *p* = 0.002). This advantage persisted for up to 24 months.

**Figure 5:**
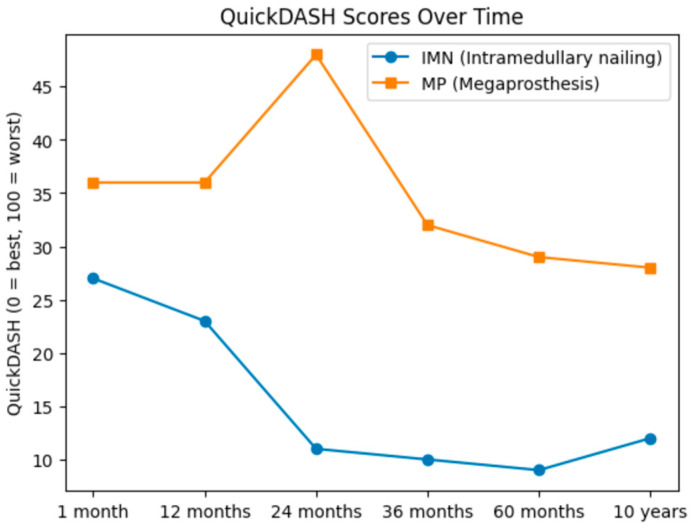
Longitudinal QuickDASH scores over time in the intramedullary nailing and megaprosthesis groups. Line graph showing patient-reported upper limb disability, with lower scores (better function) in the intramedullary nailing group during early follow-up and progressive convergence between groups at long-term evaluation.

At long-term follow-up, no statistically significant differences were observed between groups (12 vs. 28, *p* = 0.59), indicating functional convergence over time (Figure 5).

**Figure 6:**
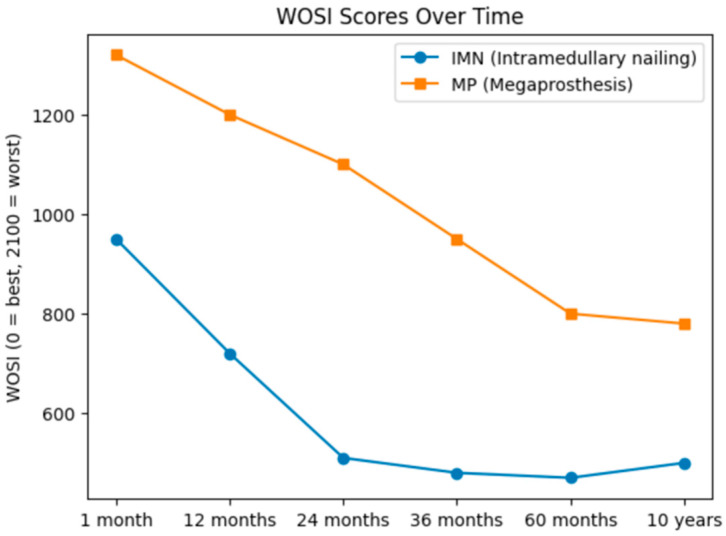
Longitudinal WOSI scores over time in the intramedullary nailing and megaprosthesis groups. Line graph illustrating shoulder-related quality of life, with significantly better early outcomes in the intramedullary nailing group and progressive convergence between groups at long-term follow-up. Lower scores indicate better function (0 = best, 2100 = worst).

The WOSI showed significantly better quality of life outcomes in the IMN group during early follow-up (*p* = 0.004). Both groups demonstrated progressive improvement over time, with no statistically significant differences at long-term evaluation (*p* = 0.61) (Figure 6).

Repeated measures ANOVA demonstrated a significant improvement over time in all functional scores (MSTS, QuickDASH, WOSI) in both groups (*p* < 0.001).

A significant interaction effect was observed in favor of the IMN group in the early postoperative phase (*p* = 0.01), confirming a faster functional recovery.

However, no significant differences in long-term functional trends were observed between the two treatment groups (*p* = 0.72).

## 4. Discussion

The management of pathological humeral fractures in oncologic patients remains a complex clinical challenge, requiring a balance between rapid functional recovery and adequate oncological control. The present study provides a direct comparison between intramedullary nailing and modular megaprosthesis, with a long-term follow-up of up to 10 years, highlighting important differences in functional recovery patterns.

Our results demonstrate that intramedullary nailing offers a significant advantage in early postoperative functional recovery, as confirmed by higher MSTS scores and lower QuickDASH and WOSI values in the first two years after surgery. These findings are consistent with previous reports emphasizing the minimally invasive nature of intramedullary fixation and its ability to provide immediate stabilization and rapid return to function [11,12].

In contrast, patients treated with megaprosthesis showed a slower initial recovery, but a progressive and sustained improvement over time, eventually reaching comparable functional outcomes at long-term follow-up. This trend reflects the more extensive nature of the surgical procedure and the need for soft tissue adaptation following tumor resection. Similar functional trajectories have been reported in oncologic reconstructions, where early impairment is followed by gradual recovery [13,14].

Importantly, our findings suggest that the choice of surgical technique does not significantly influence long-term functional outcomes but rather affects the timing of recovery. This concept is clinically relevant and supports a personalized approach to surgical decision-making. Several authors have emphasized that patient life expectancy, tumor biology, and anatomical location should guide treatment selection rather than a uniform surgical algorithm [15,16,17].

An important consideration when interpreting our findings is that the two treatment groups were not fully comparable at baseline. Intramedullary nailing was predominantly used for diaphyseal and metadiaphyseal lesions, whereas megaprosthetic reconstruction was mainly reserved for proximal humeral lesions with articular involvement and extensive bone destruction. Consequently, differences in functional recovery may partially reflect baseline anatomical and oncological characteristics rather than the surgical technique itself. This limitation is inherent to retrospective studies in orthopedic oncology, where treatment allocation is guided by clinical indications rather than randomization.

Intramedullary nailing remains particularly advantageous in patients with limited life expectancy, as it allows early mobilization and rapid pain relief with relatively low surgical morbidity. Previous studies have demonstrated that less invasive stabilization techniques are associated with shorter operative times and reduced perioperative complications, which is crucial in fragile oncologic patients [18].

On the other hand, megaprosthetic reconstruction provides superior local disease control, particularly in proximal humeral lesions with articular involvement or extensive bone destruction. The ability to perform wide tumor resection reduces the risk of local progression and mechanical failure, making megaprosthesis a reliable option in selected patients with longer expected survival [19,20].

The complication profile observed in our study was comparable between the two groups, with no statistically significant differences. However, periprosthetic infections were observed exclusively in the megaprosthesis group, reflecting the increased surgical complexity and soft tissue disruption associated with these procedures. Infection remains one of the most critical complications in oncologic reconstructions, with reported rates ranging from 5% to 15% in the literature [10,21,22].

An additional aspect deserving consideration is the potential influence of primary tumor biology on postoperative outcomes and reconstruction strategy. In the present series, no clear association was observed between the histological origin of the metastasis and the occurrence of complications, including the infectious events recorded in the megaprosthesis group. However, the limited number of complications and the relatively small cohort size precluded a reliable subgroup analysis. Despite the absence of a demonstrable correlation in our study, tumor characteristics remain an important component of treatment planning, as different malignancies exhibit distinct patterns of progression, response to systemic therapies, and expected survival. Consequently, the choice between intramedullary fixation and megaprosthetic reconstruction should continue to be individualized through a multidisciplinary evaluation that integrates anatomical factors, oncological prognosis, functional demands, and tumor-specific characteristics.

Survival analysis did not reveal significant differences between the two treatment groups, confirming that overall survival is primarily influenced by the underlying oncological disease rather than the surgical approach. This finding is consistent with previous studies demonstrating that surgical treatment in metastatic bone disease is predominantly palliative, aimed at improving quality of life rather than extending survival [5,23].

The convergence of functional outcomes at long-term follow-up represents one of the most relevant findings of this study. While intramedullary nailing ensures faster recovery, megaprosthesis allows for a gradual but sustained improvement, ultimately leading to comparable results. This observation reinforces the concept that both techniques are valid, provided that patient selection is appropriate and based on a multidisciplinary evaluation [14,24].

Our results confirm current clinical experience: intramedullary nailing provides faster functional recovery within the first 24 months, whereas megaprosthetic reconstruction offers a more durable solution for extensive proximal lesions. The key contribution of this study is the demonstration that functional scores progressively converge between the two techniques approximately 2–3 years after surgery. This finding suggests that the principal difference between the two treatment strategies lies in the speed of recovery rather than in the mid-term functional outcome. From a practical perspective, surgical decision-making should therefore be guided by patient life expectancy, oncological prognosis, and immediate functional goals rather than by the expectation of superior long-term functional results.

From a biomechanical perspective, the differences observed between the two techniques can be explained by the distinct mechanisms of load distribution and soft tissue preservation. Intramedullary devices maintain the native bone structure and muscle attachments, facilitating early function, whereas megaprosthetic reconstruction requires adaptation to an artificial joint system, which may delay recovery but ensures structural stability in cases of extensive bone loss [12,25].

Recent advances in orthopedic oncology have significantly improved outcomes through the development of modular prosthetic systems, improved fixation techniques, and enhanced perioperative management protocols. These innovations have contributed to increased implant survival and reduced complication rates, particularly in high-volume centers [7,22].

Cost-effectiveness considerations may also influence surgical decision-making. Intramedullary nailing is generally associated with lower implant costs, shorter operative times, reduced blood loss, and shorter hospital stays. In contrast, megaprosthetic reconstruction requires more expensive implants and greater surgical resources. Nevertheless, in selected patients with longer expected survival and extensive proximal humeral destruction, the durability of reconstruction and improved local disease control may justify the higher initial costs. Therefore, implant selection should balance economic considerations with oncological requirements and expected functional outcomes.

Emerging technologies may further optimize the management of these patients. Digital health tools, predictive analytics, and data-driven surgical planning have been proposed to enhance decision-making and personalize treatment strategies in orthopedic practice. Additionally, staged and multidisciplinary approaches have shown promising results in complex oncologic reconstructions, particularly in sarcoma surgery [17].

Despite these advances, the optimal management of pathological humeral fractures remains highly individualized. Factors such as tumor type, extent of bone involvement, patient comorbidities, and expected survival must all be considered when selecting the most appropriate surgical strategy.

Our study contributes to the current literature by providing long-term comparative data and reinforcing the concept that surgical decision-making should prioritize individualized patient care, balancing early functional recovery with long-term structural and oncological outcomes.

This study has several limitations that should be acknowledged. First, its retrospective design inherently exposes the analysis to selection bias, information bias, and potential inaccuracies related to incomplete medical records. Second, the relatively small sample size and single-center setting may limit the generalizability of the findings. Third, treatment allocation was not randomized, as intramedullary nailing was primarily used for diaphyseal lesions, whereas megaprosthetic reconstruction was generally reserved for proximal lesions with articular involvement and extensive bone loss. Consequently, differences in postoperative recovery may partially reflect baseline anatomical and oncological differences rather than the surgical technique itself. Finally, the long follow-up period introduces the possibility of survivor bias, since patients with more favorable oncological prognoses were more likely to contribute data at later follow-up intervals. Furthermore, long-term functional analyses should be interpreted with caution. Only 20 patients remained available for assessment at five years and only 4 patients at ten years. Consequently, late outcome measurements may be affected by survivor selection bias, as patients with more favorable oncological prognoses were more likely to contribute long-term data. Therefore, the results should be interpreted with caution and confirmed through prospective multicenter studies with larger patient cohorts.

## 5. Conclusions

Both intramedullary nailing and modular megaprosthesis represent effective surgical strategies for the treatment of pathological and impending humeral fractures in oncologic patients. Intramedullary nailing appears to provide faster early functional recovery, whereas megaprosthetic reconstruction offers a durable reconstructive option for patients with extensive proximal humeral involvement.

Importantly, the principal finding of this study is that functional outcomes progressively converge between the two techniques approximately 2–3 years after surgery. This suggests that the main difference between the two approaches lies in the speed of recovery rather than in the mid-term functional outcome. Mid-term results up to five years appear comparable between the two techniques, although long-term comparisons should be interpreted with caution because of the limited number of surviving patients available for evaluation at ten years.

Therefore, surgical decision-making should remain individualized and based on lesion characteristics, tumor biology, expected survival, functional requirements, and multidisciplinary evaluation, with the goal of optimizing both oncological control and postoperative quality of life.

A multidisciplinary approach remains essential to optimize both oncological and functional outcomes.

## Figures and Tables

**Table 1:** Baseline demographic and clinical characteristics of the study population (*n* = 48).

Variable	Value
Total patients, *n*	48
Age, mean (range), years	66 (41–87)
Male sex, *n* (%)	19 (39.6)
Female sex, *n* (%)	29 (60.4)
Pathological fractures, *n* (%)	45 (93.7)
Impending fractures (Mirels ≥ 9), *n* (%)	3 (6.3)
Intramedullary nailing (IMN), *n* (%)	26 (54.2)
Megaprosthesis (MP), *n* (%)	22 (45.8)
Multiple myeloma with multiple skeletal lesions, *n* (%)	2 (4.2)

**Table 2:** Postoperative complications in the intramedullary nailing and megaprosthesis groups. Distribution of neurological, infectious, and mechanical complications, with comparison between groups. *p*-values were calculated using chi-square or Fisher’s exact test, as appropriate. N.S.: not significant.

Complication	Intramedullary Nailing (*n* = 26)	Megaprosthesis (*n* = 22)	Total (*n* = 48)	*p*-Value
Sensory deficits	2 (7.7%)	2 (9.1%)	4 (8.3%)	0.99
Radial nerve palsy	2 (7.7%)	0 (0%)	2 (4.1%)	0.49
Overall neurological complications	4 (15.4%)	2 (9.1%)	6 (12.5%)	0.67
Periprosthetic infections	0 (0%)	2 (9.1%)	2 (4.1%)	0.21
Mechanical failures	3 (11.5%)	0 (0%)	3 (6.3%)	N.S.
Dislocations	0 (0%)	5 (22.7%)	5 (10.4%)	N.S.
Local recurrence	0 (0%)	0 (0%)	0 (0%)	N.S.

## Data Availability

The datasets generated and/or analyzed during the current study are available from the corresponding author on reasonable request.

## Abbreviations

The following abbreviations are used in this manuscript:

IMN	Intramedullary Nailing
MP	Megaprosthesis
MSTS	Musculoskeletal Tumor Society
QuickDASH	Quick Disabilities of the Arm, Shoulder and Hand
WOSI	Western Ontario Shoulder Instability Index
SD	Standard Deviation
IRB	Institutional Review Board
